# Genomic analyses support locally derived crown-of-thorns seastar outbreaks in the Pacific

**DOI:** 10.1186/s12915-025-02350-4

**Published:** 2025-08-06

**Authors:** Carlos Leiva, Marta Martín-Huete, Sarah Lemer

**Affiliations:** 1 Marine Laboratory, University of Guam, 303 University Drive, Mangilao, Guam 96923 USA; 2 Estación Biológica de Doñana (EBD-CSIC), C/Americo Vespucio 26, Seville, 41092 Spain; 3 Department of Evolutionary Biology, Ecology & Environmental Sciences, Faculty of Biology, University of Barcelona, Av. Diagonal 643, Barcelona, 08028 Spain; 4 Institut de Recerca de La Biodiversitat (IRBio), Faculty of Biology, University of Barcelona, Barcelona, Spain; 5 Museum of Nature, Leibniz Institute for the Analysis of Biodiversity Change, Martin-Luther-King-Platz 3, Hamburg, 20146 Germany

**Keywords:** *Acanthaster*, Population genomics, Phylogenomics, COTS, Coral reefs

## Abstract

**Background:**

Crown-of-thorns seastars (COTS, *Acanthaster* spp.) are the most notorious coral predators, whose devastating outbreaks cause recurrent and extensive coral depletion across Indo-Pacific reefs. However, the spread potential of COTS outbreaks and the anthropogenic role in their initiation have remained a subject of intense debate for over five decades.

**Results:**

Here, using low-coverage whole-genome sequences of 247 COTS, we show that Pacific COTS populations are highly structured, indicating that outbreaks do not spread through open ocean, but instead are locally derived. Pacific COTS populations are grouped in three main lineages geographically restricted to Hawai‘i, French Polynesia, and the West Pacific, with the latter showing further significant genetic substructure. Phylogenomic analyses indicated that the Hawai‘i COTS lineage likely represents a different undescribed species and challenged the species status of both *A.* cf. *solaris* and the Eastern Pacific COTS species (*A. ellisii*), as the latter appeared as the sister group of the French Polynesia COTS lineage. Additionally, we show that current COTS populations present the highest effective sizes of the last million years, suggesting that human and/or climate change may influence COTS population sizes.

**Conclusions:**

Overall, our study highlights the improvements brought by low-coverage whole-genome sequencing approaches in resolving the phylogeny and connectivity patterns of a keystone species in understudied regions of the Pacific Ocean.

**Supplementary Information:**

The online version contains supplementary material available at 10.1186/s12915-025-02350-4.

## Background

Coral reefs represent the pinnacle of marine biodiversity: despite covering less than 0.2% of the seafloor, they host a remarkable one-third of all known marine species [1]. Additionally, they provide essential ecosystem services to coastal communities and economies, including coastal protection, cultural values, food security, and revenue from tourism [2]. However, coral reefs face significant threats from anthropogenic climate change, which is leading to more frequent and severe bleaching and mortality events caused by marine heatwaves [3].

Together with widespread bleaching events, outbreaks of the coral-eating crown-of-thorns seastars (COTS, *Acanthaster* species) remain a major cause of coral reef degradation in the Indo-Pacific, well documented since the 1960s [4]. Originally considered a single species under the name of *Acanthaster planci*, five different COTS species with geographically restricted distributions are currently accepted [5, 6]. COTS outbreaks are commonly characterized by a sudden and major increase in population size, reaching density levels that the reef ecosystem cannot sustain. Larval, juvenile, and adult feeding behaviors have been thoroughly studied (e.g., [7–9]), as they all have relevant implications in COTS outbreaks. In numerous reefs, the impact of COTS outbreaks has even surpassed the cumulative effects of all other significant disturbances (e.g., [10, 11]). Indeed, COTS are the most destructive coral predators [12], which have the potential of rapidly altering community composition and structure, and promoting algal colonization [4, 13]. In reefs with low human pressures, COTS impacts on coral communities are generally deemed negligible [14]. However, their devastating predation pressure teams up with the unprecedented marine heatwaves, overfishing, high sedimentation, and eutrophication that most coral reefs currently experience, further degrading vital coral reef ecosystems [14].

Despite being among the most studied reef-associated taxa, important questions about COTS biology, ecology, and management still remain unresolved [15]. One central question that has created persistent controversy is whether COTS outbreaks are natural events that went unnoticed before the 1960s, or if they are otherwise influenced by the recent degradation of coral reefs due to anthropogenic impacts [15]. Research from coral core data, laboratory experiments on COTS larvae, and model simulations seem to indicate an increase in severity and extension of COTS outbreaks in the recent past [14, 16], suggesting an anthropogenic role in COTS abundance and outbreaks. The “terrestrial run-off hypothesis” suggests that increased river nutrient loads provoke the phytoplankton blooms *Acanthaster* larvae feed on, resulting in increased COTS outbreaks [17–19]. However, the most recent hypothesis linking human activities to COTS outbreaks, the “degraded reef hypothesis”, suggests that coral reef degradation benefits COTS juveniles as they accumulate in coral rubble before their pulsed emergence as coral-eaters, which also explains the episodic nature of COTS outbreaks through this negative feedback loop [20].

Another open question is the dispersal capacity of COTS larvae and, ultimately, of COTS outbreaks. If COTS outbreaks are spreading from one reef to another through open ocean (i.e., “secondary outbreaks” hypothesis [21]), little can be done locally to avoid or control them. On the other hand, if COTS outbreaks are localized events driven by an increase in local larvae only [21], management measures can be put in place to avoid them or minimize their impacts. This topic has been extensively explored during the last four decades using population genetics approaches in the Pacific COTS species, *Acanthaster* cf. *solaris*. However, different studies using different genetic markers reached opposite conclusions. First, high connectivity throughout the Pacific Ocean leading to admixed populations was supported using allozymes [22] and microsatellites [23, 24]. Later on, other studies detected structured populations suggesting regionally derived outbreaks, using the highly variable mitochondrial DNA control region [25, 26]. However, more recently, the analysis of complete mitochondrial genomes pointed once again to high connectivity and admixed populations throughout the Pacific, with a Pan-Pacific clade having a potentially high dispersal ability [27]. In order to assemble mitochondrial genomes, Yasuda and collaborators sequenced COTS genomes at a mean depth of 10X, but these nuclear genome data were not actually analyzed [27].

Here, our main aim was to disentangle this conundrum regarding COTS outbreak dispersal potential, using publicly available nuclear low-coverage whole-genome sequence data of Pacific COTS, *A.* cf. *solaris*, throughout their distribution range—using the low-coverage whole-genome sequence raw data that Yasuda and collaborators sequenced and used to assemble mitochondrial genomes, but did not analyze [27]. Additionally, we used demographic history models to test whether genomic data support an anthropogenic influence on COTS outbreaks. If so, we would expect to see recent increases in the effective population sizes of Pacific COTS populations. Finally, in order to place the Pacific COTS lineages found here in their phylogenetic context, we used whole genome and transcriptome resources from other *Acanthaster* species to resolve the still ambiguous phylogenetic relationships among all COTS species and lineages sequenced to date.

## Results

The data downloaded from the NCBI SRA BioProjects PRJDB10499, PRJDB9937, and PRJNA548418 consisted of a total of 12,011.8 million reads for 241 *Acanthaster* cf. *solaris*, three *A.* cf. *ellisii*, two *A. benziei*, two *A. planci*, and one *A. brevispinus*, with an average of 48.4 million reads per individual (Additional file 1: Table S1). A total of 7914.4 million clean reads mapped to the *A.* cf. *solaris* reference genome [28] and passed post-mapping filters, with an average of 32.0 million reads per sample (Additional file 1: Table S1). The initial average depth across samples was 8.3 ×, which increased to 9.5 × after removing low-coverage samples that were not used in subsequent analyses (Additional file 1: Table S1).

### Three main lineages of *A.* cf. *solaris* with lack of open-ocean connectivity

Population genomic analyses were performed on a dataset of 198 *A.* cf. *solaris* individuals and 7.1 million genome-wide SNPs shared by at least 80% of the samples, after excluding samples with low coverage and contaminated samples (Additional file 1: Table S1 and Additional file 2: Table S2). *Acanthaster* cf. *ellisii* samples from the Gulf of California were excluded from this dataset as they presented extremely low coverage (0.3–1.3 ×), which would have introduced depth biases in population genomic analyses.

**Fig. 1:**
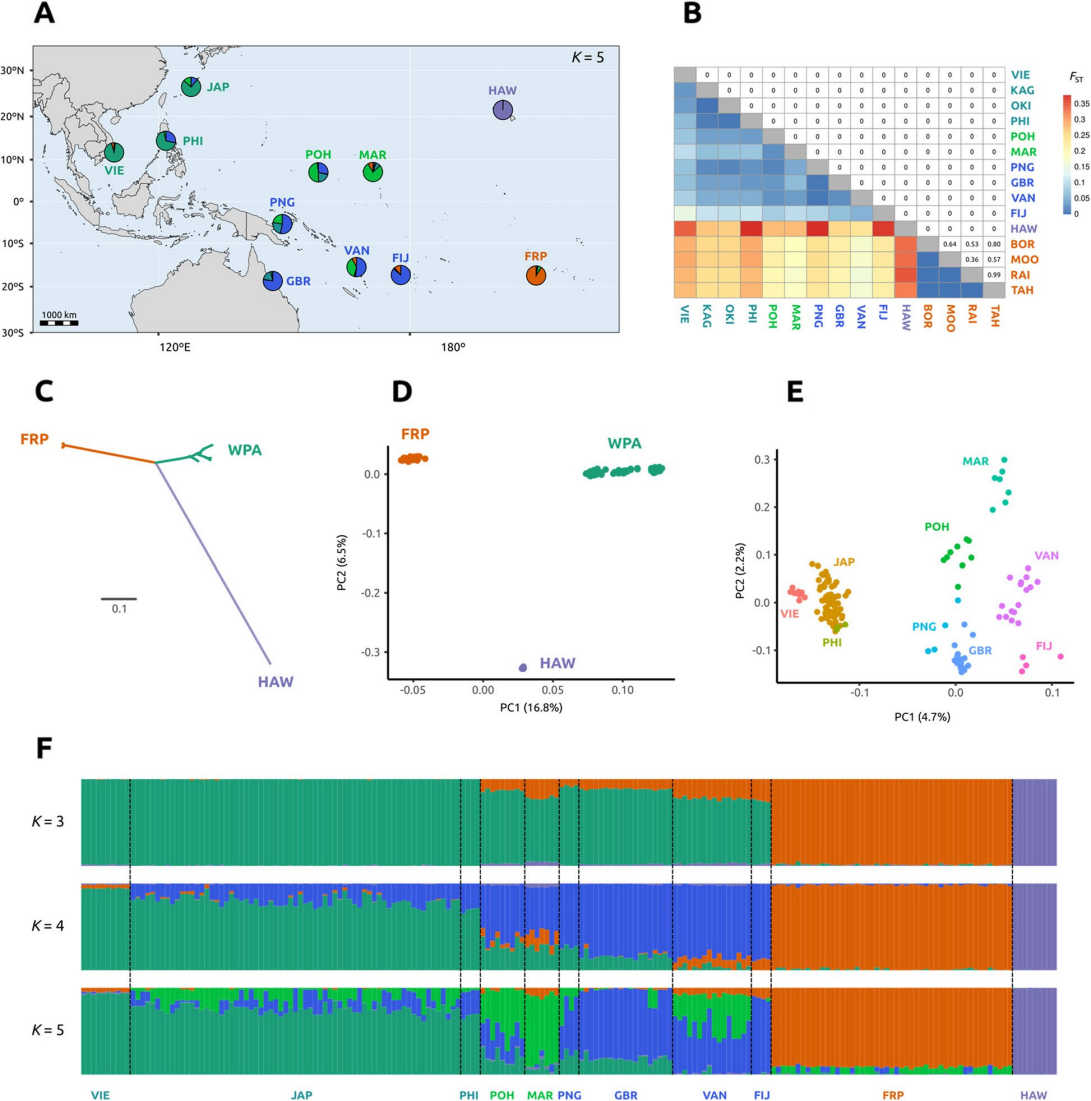
Fine population structure in Pacific COTS. **A** Study area with pie charts representing admixture results with *K* = 5. Sampling sites abbreviations: Vietnam (VIE), Philippines (PHI), Japan (JAP), Pohnpei (POH), Marshall Islands (MAR), Papua New Guinea (PNG), Great Barrier Reef (GBR), Vanuatu (VAN), Fiji (FIJ), French Polynesia (FRP), Hawai‘i (HAW). **B** Pairwise *F*_ST_ table showing differentiation between pairs of sampling sites. Heatmap in lower diagonal represents the *F*_ST_ values, with the upper diagonal showing their associated *p*-values. Sampling sites in Japan and French Polynesia were analyzed independently for this analysis: Okinawa (OKI), Kagoshima (KAG), Bora Bora (BOR), Mo‘orea (MOO), Raiatea (RAI), Tahiti (TAH). **C** Neighbor-joining tree and **D** principal component analysis (PCA), showing the split of the samples in three main lineages: West Pacific (WPA, teal), French Polynesia (FRP, orange) and Hawai‘i (HAW, purple). **E** PCA results using the samples from the WPA lineage only. Abbreviations following **A**. **F** Admixture results for *K* = 3, *K* = 4 and *K* = 5. Abbreviations following **A**

**Fig. 2:**
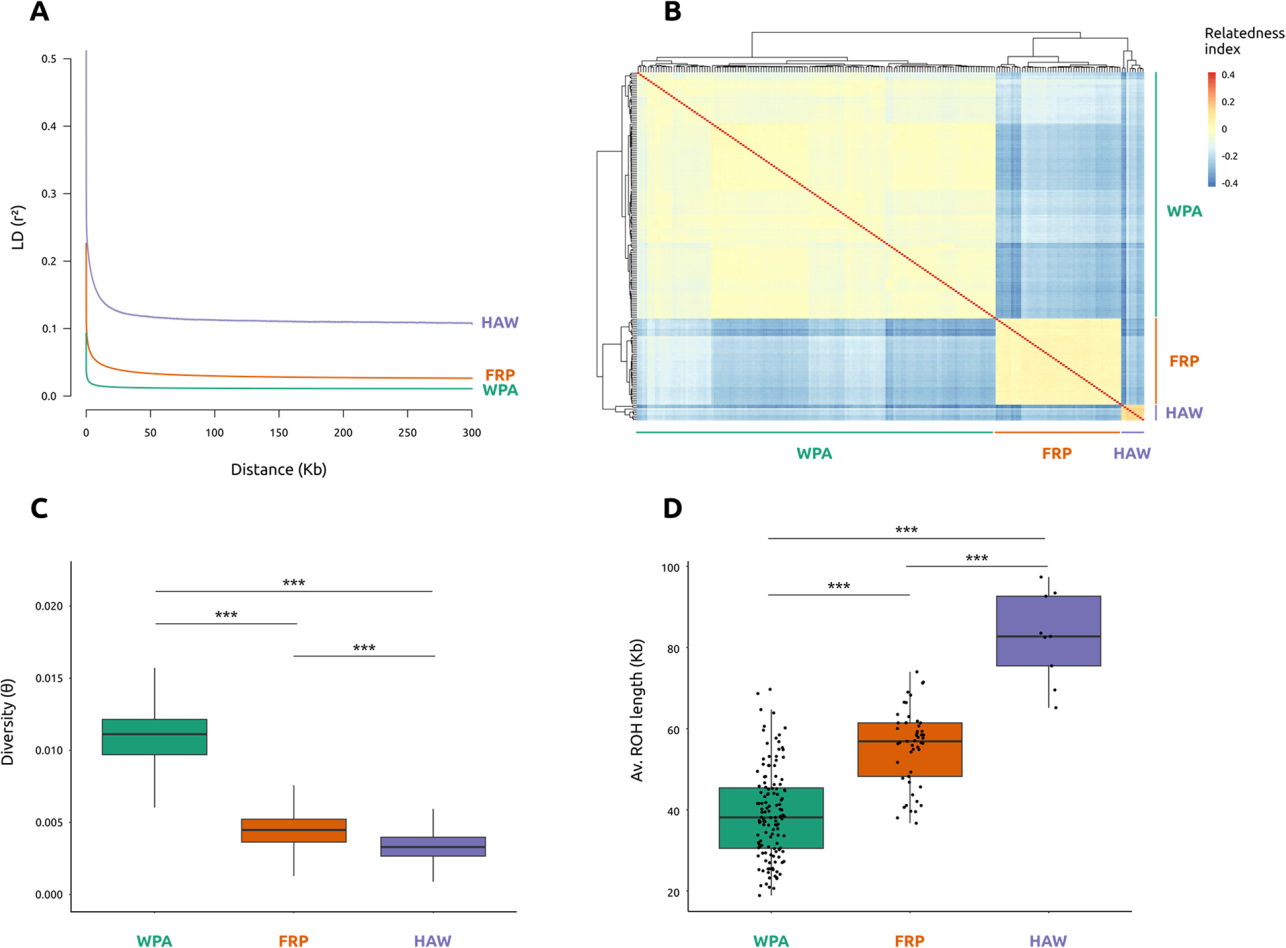
Diversity and inbreeding measures of Pacific COTS lineages.** A** Linkage disequilibrium (LD) decay measured as the square of the correlation coefficient between pairs of loci (*r*^2^). **B** Relatedness indices between pairs of samples. **C** Genetic diversity for each lineage, measured as thetas (θ) in non-overlapping sliding windows of 50,000 base pairs. Significant differences were found between all pairwise comparisons (Mann–Whitney *U* test, *p*-values < 1e^−16^), represented by three asterisks. **D** Comparison of average ROH length per individual, grouped by lineage. Significant differences were found between all pairwise comparisons (Mann–Whitney *U* test, *p*-values < 1e^−4^), represented by three asterisks

Population structure analyses were performed on a thinned dataset of 38,260 independent SNPs with a minimum distance of 10,000 bp between SNPs in order to ensure the use of independent markers, which is a requirement for population structure analyses. Population structure results detected three main lineages: Hawai‘i, French Polynesia, and West Pacific (Fig. 1). This was supported by all our analyses, including the pairwise *F*_ST_ indices (Fig. 1B), a neighbor-joining tree (Fig. 1C), a principal component analysis (PCA) (Fig. 1D), admixture analyses (Fig. 1F), and relatedness analyses (Fig. 2B). Moreover, these three main lineages presented notable differences in diversity and inbreeding (Fig. 2). The Hawai‘i lineage featured genomic signatures of high inbreeding, with the highest LD values in the LD decay curves (Fig. 2A), the highest relatedness index values (Fig. 2B) and significantly longer runs of homozygosity (ROH) (Fig. 2D) (pairwise Mann–Whitney *U* test, *p*-values < 1e^−4^). This was accompanied by having significantly lower genetic diversity (theta, θ) than the other two lineages (Fig. 2C) (pairwise Mann–Whitney *U* test, *p*-values < 1e^−16^). On the other hand, the West Pacific lineage presented the lowest LD values in the LD decay curves (Fig. 2A), significantly shorter ROH (Fig. 2D) (pairwise Mann–Whitney *U* test, *p*-values < 1e^−5^) and significantly higher genetic diversity (theta, θ) (Fig. 2C) (pairwise Mann–Whitney *U* test, *p*-values < 1e^−16^). The French Polynesia lineage presented intermediate values in all diversity and inbreeding analyses.

Further population structure analyses focusing on the West Pacific lineage revealed limited open-ocean connectivity between neighboring regions. PCA results showed that samples from the same sampling site tend to group together in a distribution that mimics the geographical position of our sampling sites (Fig. 1E), indicating a lack of open-ocean connectivity of COTS populations. The ordination of samples on the first PCA axis followed the longitudinal position of the sampling sites in the study area (Fig. 1E). Besides revealing the three main lineages, admixture results using the whole dataset were in agreement with this geographic structuring of the West Pacific lineage, showing a West-to-East gradient with *K* = 4 (Fig. 1F). Admixture results with *K* = 5 split the West Pacific lineage into three genetic clusters (Fig. 1A and F): (i) West Pacific–Asia, which is predominant in Vietnam (VIE), Japan (JAP), and the Philippines (PHI); (ii) West Pacific–Micronesia, prevailing in Pohnpei (POH) and the Marshall Islands (MAR); and (iii) West Pacific–Southwest, which is the most common genetic cluster in Papua New Guinea (PNG), the Great Barrier Reef (GBR), Vanuatu (VAN), and Fiji (FIJ). Additionally, admixture results showed that adjacent sampling sites such as Pohnpei (POH) and the Marshall Islands (MAR) or Vanuatu (VAN) and Fiji (FIJ) present differences in their admixture proportions, suggesting significant genetic differences between these neighboring island pairs (Fig. 1A and F). Pairwise *F*_ST_ measures showed that, while genetic differences were relatively small between all pairs of sampling sites in the West Pacific, all of them were statistically significant (1000 bootstrap replicates, 95 confidence intervals, all *p*-values = 0) (Fig. 1B). Pairwise *F*_ST_ values were also statistically significant even between the two sampling sites in Japan, Okinawa (OKI) and Kagoshima (KAG), which were analyzed independently for this analysis. Contrastingly, there were no significant differences among the four sampling sites in French Polynesia: Bora Bora (BOR), Mo‘orea (MOO), Raiatea (RAI) and Tahiti (TAH) (Fig. 1B).

### Different selective pressures among Pacific COTS lineages

**Fig. 3:**
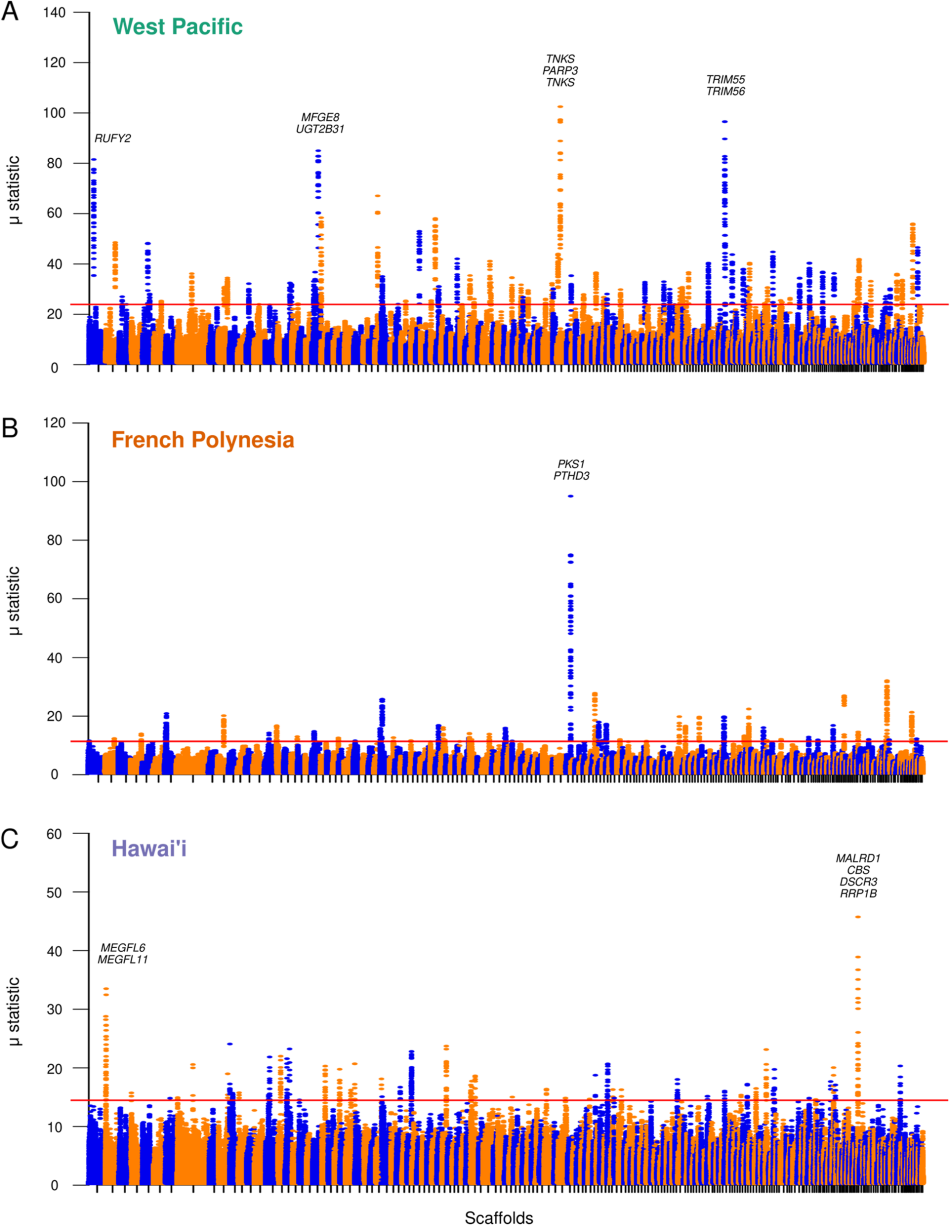
Selective pressures in Pacific COTS lineages. Manhattan plot of the μ statistic for each lineage: **A** West Pacific, **B** French Polynesia, and **C** Hawai‘i. Dots represent genomic windows and are colored by scaffold (odd scaffolds are blue, even scaffolds are orange). Red horizontal lines represent the 99.95 percentile. Gene symbols for the genes included in the top windows for each lineage are also shown in the plots (see Table 1 for more details)

In order to test whether the separation of the three *A.* cf. *solaris* lineages was merely due to neutral processes (oceanographic fronts, connectivity, genetic drift) or if selection was also involved, we looked for genomic signals of hard selective sweeps in each Pacific COTS lineage. RAiSD results showed different genes and genomic regions presenting signals of selective sweeps in each lineage (Fig. 3; Table 1). In the West Pacific, four genomic regions located in scaffolds 1, 21, 64, and 111 (totaling 8 genes) had strong signals of hard selective sweeps (Fig. 3A; Table 1). Contrastingly, in French Polynesia, a single genomic region stood out with strong signals of a selective sweep, in scaffold 65, which included two genes (Fig. 3B; Table 1). Finally, in Hawai‘i, two genomic regions presented strong signals of hard selective sweeps, in scaffold 166 (four genes) and scaffold 2 (two genes) (Fig. 3C; Table 1). Genes presenting signals of selective sweeps play key roles in development, immunity, and metabolism (Table 1), highlighting their adaptive relevance in each COTS lineage.

**Table 1:** Hard selective sweeps in the Pacific COTS lineages. For each selective sweep, details include the scaffold, windows, μ statistic, and genes within the selected region, together with gene functions

Lineage	Scaffold number	Scaffold name	Window starts	Window ends	μ statistic	Gene symbol	Gene name
West Pacific	1	NW_019091355	1,628,693	1,673,935	81.51	RUFY2	RUN and FYVE domain-containing Protein 2
	21	NW_019091375	2,270,703	2,351,916	85	MFGE8	Lactadherin
						UGT2B31	UDP-glucuronosyltransferase 2B31-like
	64	NW_019091418	1,261,426	1,337,030	102.5	TNKS	Tankyrase
						TNKS	Tankyrase
						PARP3	Poly [ADP-ribose] polymerase 3
	111	NW_019091465	400,759	496,273	96.58	TRIM55	Tripartite motif-containing protein 55
						TRIM56	E3 ubiquitin-protein ligase TRIM56
French Polynesia	65	NW_019091419	1,682,033	1,737,827	95.03	PKS1	Reducing polyketide synthase PKS1
						PTCHD3	Patched domain-containing protein 3
Hawai‘i	2	NW_019091356	215,805	299,107	33.5	MEGFL6	Multiple epidermal growth factor-like domains protein 6
						MEGFL11	Multiple epidermal growth factor-like domains protein 11
	166	NW_019091520	130,050	184,656	45.74	MALRD1	MAM and LDL-receptor class A domain-containing protein 1
						CBS	Cystathionine beta-synthase
						DSCR3	Down syndrome critical region protein 3 homolog
						RRP1B	Ribosomal RNA processing protein 1 homolog B

### The current Ne of Pacific COTS populations is the highest in the last million years

**Fig. 4:**
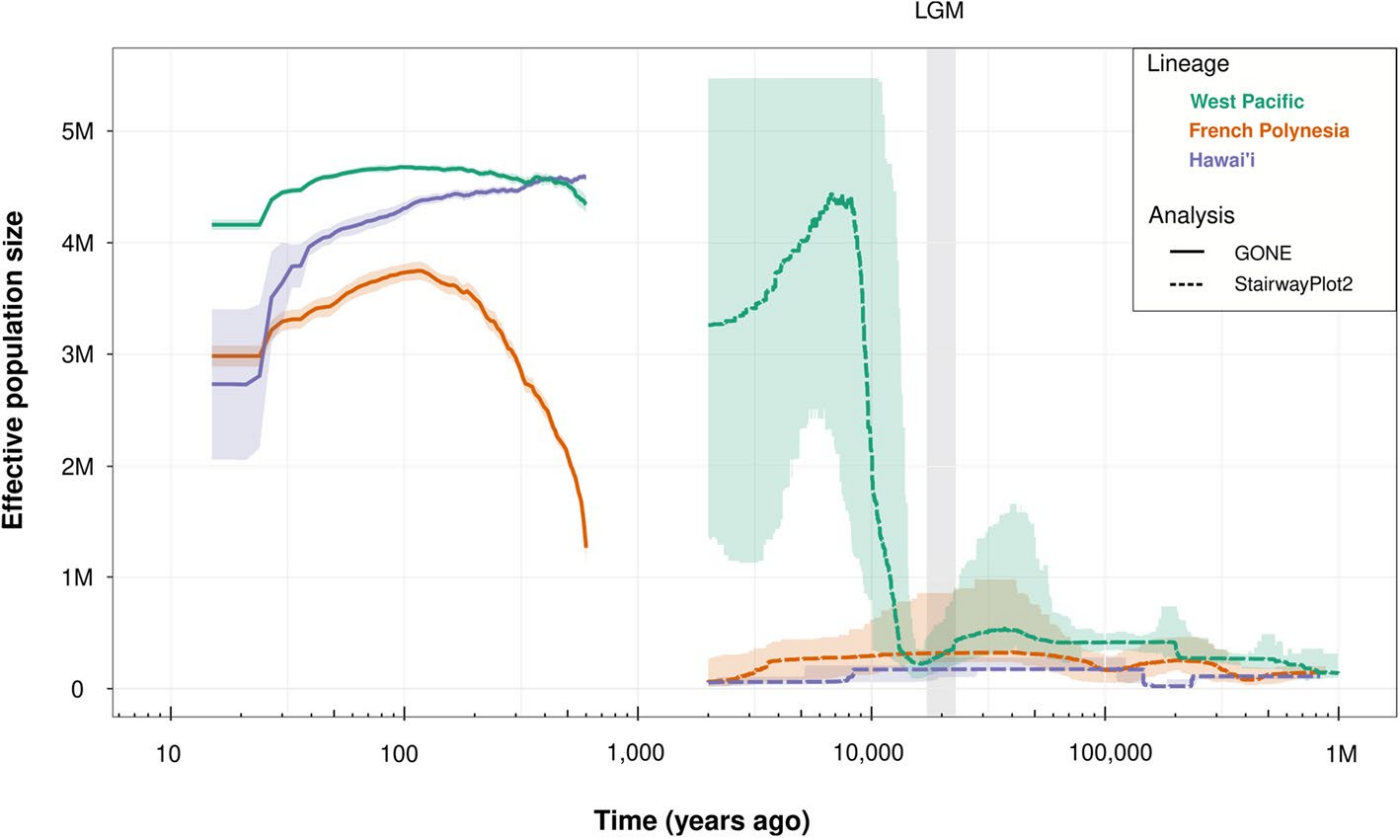
Demographic history of Pacific COTS lineages across the last million years. Temporal reconstruction of effective population sizes was estimated with the software GONE (straight line, left) and StairwayPlot2 (dashed line, right) for each Pacific COTS lineage: West Pacific (teal), French Polynesia (orange) and Hawai‘i (purple). Shaded colored areas represent the 95% confidence intervals. Vertical shaded gray area represents the Last Glacial Maximum (LGM)

To understand the demographic history of Pacific COTS populations and infer the potential anthropogenic effect on their current outbreaks, we estimated changes in effective population size (*N*e) through time for each *A.* cf. *solaris* lineage. Ancient (StairwayPlot2) and recent (GONE) demographic history results did not overlap, resulting in a data gap between 2000 and 600 years ago (Fig. 4). Ancient demographic history results from StairwayPlot2 showed that since one million years ago up to the last glacial maximum (LGM), the three lineages maintained a relatively stable and small *N*e, lower than 500,000 individuals (Fig. 4). During this whole period, the West Pacific lineage presented a slightly higher *N*e than the other two lineages, with Hawai‘i showing the smallest *N*e values (Fig. 4). More recently, at the onset of the Holocene, around 12,000 years ago, the West Pacific lineage experienced a rapid population expansion, surpassing 4 million individuals (Fig. 4).

Recent *N*e of the three lineages (from 600 years ago to current) all appear strikingly higher than their respective ancient *N*e values. Specifically, the West Pacific lineage maintained a high and constant *N*e during the last 600 years, above 4 million individuals (Fig. 4). The Hawai‘i lineage suffered a decrease of *N*e, from 4.6 million individuals to 2.7 million individuals (Fig. 4). Finally, the French Polynesia lineage experienced a population expansion from 600 years ago to 100 years ago, peaking at 3.7 million individuals before experiencing a slight reduction to its current size of 3 million individuals (Fig. 4). Despite these fluctuations, our results clearly showed that the *N*e of the Pacific COTS populations have recently reached their highest values of the last million years, especially in French Polynesia and Hawai‘i (Fig. 4).

### *Acanthaster* cf. *solaris* is not a valid monophyletic species

We used a phylogenomic approach in order to explore the relationships between the different COTS species and lineages: *A. benziei*, *A. planci*, *A.* cf. *solaris* (with its three lineages: West Pacific, French Polynesia and Hawai‘i), and *A.* cf. *ellisii*. Our dataset consisted of 204 COTS individuals from four nominal species and 4.6 million genome-wide SNPs shared by at least 90% of the samples. Additionally, to properly root the tree, we used transcriptomic data from *Acanthaster brevispinus*, with a dataset only including a representative of each COTS species and lineage. This dataset included 8 individuals and 46,438 genome-wide SNPs shared by all samples, and the maximum likelihood tree robustly supported *A. benziei* and *A. planci* as sister species (Additional file 3: Fig. S1).

**Fig. 5:**
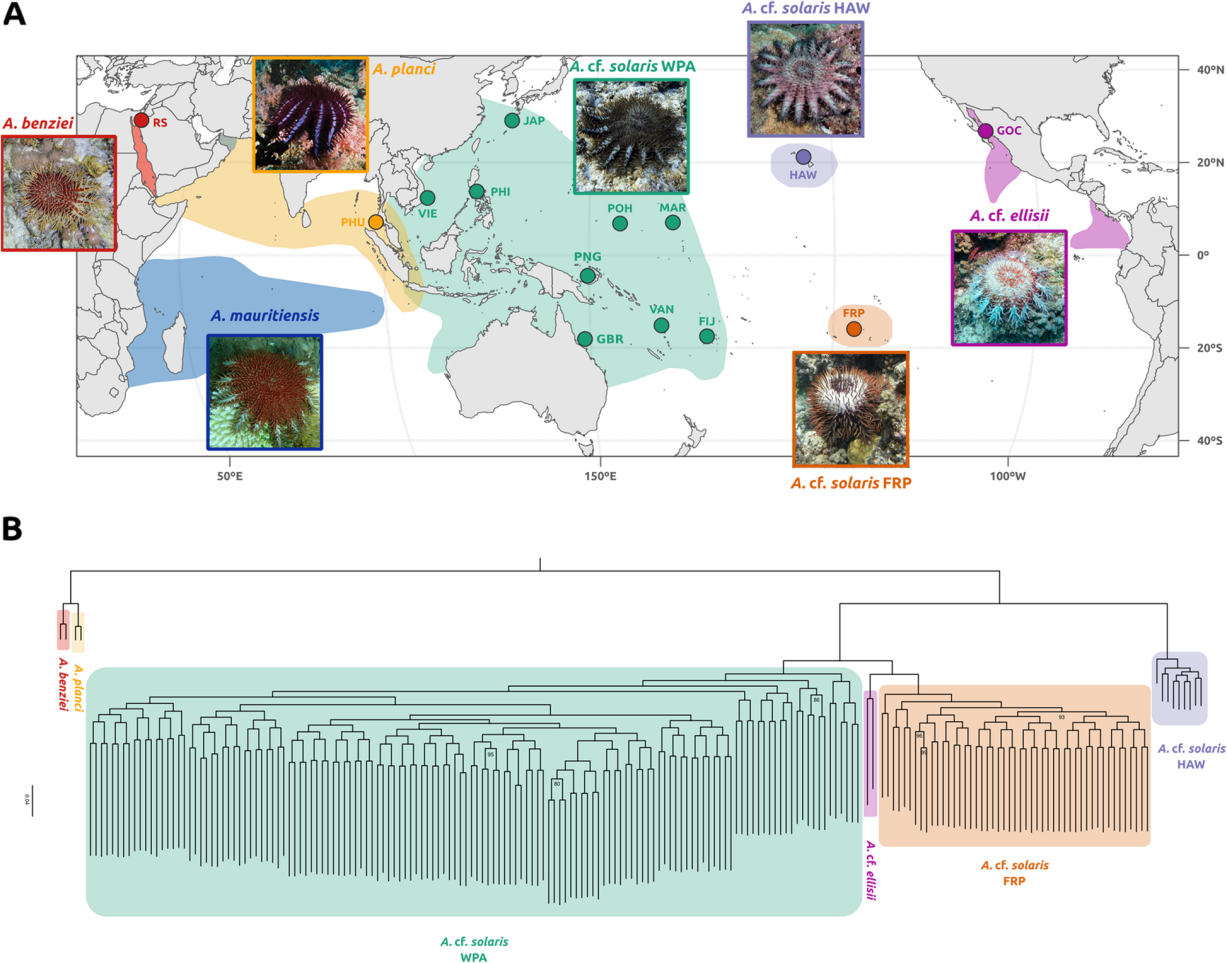
Phylogenomic hypothesis for all sequenced COTS species and lineages. **A** Map of the Indian and Pacific Oceans showing the distribution of each COTS species and lineage, and the sites of the samples used for the phylogenomic analyses. Distributions based on Uthicke and collaborators [5] and our results. Pictures retrieved from iNaturalist: *A. mauritiensis* from “mwanaisha_musa” (https://www.inaturalist.org/observations/217679285), *A. benziei* from “cleopatrabrowne” (https://www.inaturalist.org/observations/124175872), *A. planci* from “dennisthediver” (https://www.inaturalist.org/observations/71364104), *A.* cf. *solaris* WPA from “marisa_a” (https://www.inaturalist.org/observations/107288185), *A*. cf. *solaris* FRP from “andrea_glockner” (https://www.inaturalist.org/observations/11250240), *A.* cf. *solaris* HAW from “davidr” (https://www.inaturalist.org/observations/55365598), *A.* cf. *ellisii* from “adnovo” (https://www.inaturalist.org/observations/206922721). Sampling sites abbreviations following Fig. 1A; additionally: Red Sea (RS), Phuket (PHU), Gulf of California (GOC). **B** Maximum likelihood tree for all COTS species and lineages. Only bootstrap values different than 100 are shown

The maximum likelihood tree for the 204 COTS samples revealed four highly supported and geographically restricted COTS lineages in the Pacific Ocean (Fig. 5B). The nine *A.* cf. *solaris* individuals from Hawai‘i formed an isolated clade sister to all other Pacific COTS samples (Fig. 5B). The other *A.* cf. *solaris* samples were split into three clades: a French Polynesian clade, which appeared sister to the clade formed by *A.* cf. *ellisii* from the Gulf of California (GOC), and a clade containing all *A.* cf. *solaris* from the West Pacific, sister to the French Polynesian and *A.* cf. *ellisii* clades (Fig. 5B).

## Discussion

In the present study, we expand our understanding of *Acanthaster* evolution, Pacific COTS outbreaks propagation, and their recent demographic history. Our results provide unequivocal genomic evidence to indicate that (i) COTS outbreaks do not spread through open ocean waters in the Pacific Ocean; and (ii) current Pacific COTS populations present their highest effective population sizes (*N*e) of the last million years, with a recent steep increase in French Polynesia and Hawai‘i suggesting anthropogenic influence.

Contrasting with the previously published mitogenome results [27], our low-coverage whole-nuclear genome sequencing analyses show that *A.* cf. *solaris* populations are structured in three main lineages (West Pacific, French Polynesia and Hawai‘i), with fine-scale structure within the West Pacific lineage. The fact that this is in agreement with the previous results from the mitochondrial control region [25, 26] but not with the whole mitogenome results [27] suggests that there is incomplete lineage sorting in most of the mitochondrial genome. This lack of connectivity among Pacific regions is a common pattern in reef-associated taxa, including echinoderms [29], and refutes the previously accepted idea that high larval dispersal promotes the spread of COTS outbreaks [27, 30]. Instead, our results show a lack of open-ocean connectivity among the different sampling sites of the West Pacific, indicating that COTS outbreaks are locally derived in the West Pacific, i.e., the increase in adults during outbreaks originates exclusively from local larvae. Remarkably, we report significant genetic differences even between neighboring regions, such as between Okinawa and Kagoshima (only ~ 750 km apart). These results contradict the lack of genetic differentiation previously found in that same geographic region using reduced representation sequencing approaches [31, 32], which may be explained by the low number of SNPs analyzed by these studies, only 186 and 115 SNPs. The low-coverage whole-nuclear genome sequence dataset analyzed here demonstrates that the “secondary outbreak” hypothesis, which has only been well documented in the Great Barrier Reef [4], is an exception to the rule of independent locally derived outbreaks. However, for French Polynesia, we cannot rule out the “secondary outbreak” hypothesis, as no genetic differences were found among COTS populations from the four sampled islands.

Our phylogenetic results supported the population structure results and challenged the current species status of *A.* cf. *solaris* and *A.* cf. *ellisii*. Most recent studies of the COTS species complex conducted with limited genetic data agreed on the presence of five species, including *A. benziei* limited to the Red Sea, *A. planci* and *A. mauritiensis* in the Indian Ocean, *A.* cf. *ellisii* in the Eastern Pacific, and *A.* cf. *solaris* as a widespread species in the rest of the Pacific Ocean [5, 33]. However, our results clearly indicate that Hawai‘i COTS formed a clade with a relatively long branch, sister group of all the rest of Pacific COTS samples, including the *A.* cf. *ellisii* individuals from the Eastern Pacific. This differentiation of the Hawai‘i COTS is in agreement with the previously published mitogenome phylogeny [27], and strongly suggests that Hawai‘i COTS belong to a different yet to be described species. For the remaining Pacific COTS lineages—excluding the highly differentiated clade from Hawai‘i—our results point to two possible systematic outcomes: they could all be grouped under a single species, named *A.* cf. *solaris*, which would invalidate *A.* cf. *ellisii*; or they could be split into three different species: *A.* cf. *ellisii* in the Eastern Pacific, *A.* cf. *solaris* in the West Pacific, and a new COTS species in French Polynesia. Our diversity, inbreeding, and selective sweeps results for three of these four Pacific COTS lineages illustrated the differences among them and highlighted the intrinsic features that characterize each lineage. Similar differences were previously found in cryptic speciation events and adaptive radiations [34, 35], emphasizing the need of a thorough systematic review of *Acanthaster* spp. in the Pacific Ocean.

The combination of different analytical approaches yielded an almost complete demographic history reconstruction of the last million years for three Pacific COTS lineages. The West Pacific lineage displayed a population expansion coinciding with the onset of the Holocene, starting around 12,000 years ago. Similar expansions after the Last Glacial Maximum (LGM) are frequent in marine invertebrate taxa, including other echinoderms (e.g., [36, 37]). In the oceanographically isolated French Polynesia and Hawai‘i regions, however, COTS demographic histories were not affected by the post-LGM deglaciation, with both lineages showing a much more recent population expansion. Unfortunately, the precise starting time of these expansions is difficult to know with the current data, as it lays on the low confidence windows for both demographic history analyses performed. A chromosome-level reference genome for *Acanthaster* may improve these estimations in the future. Despite this limitation, our results indicate that population expansions started between 2000 and 600 years ago, potentially suggesting an anthropogenic role in the current COTS populations. This potentially human-mediated demographic expansion could be due to the increased fishing pressure—as fish biomass removal is a known driver of COTS outbreaks [38]—or due to coral reef degradation in line with the degraded reef hypothesis [21].

Our results are inconsistent with the hypothesis that high larval dispersal explains the propagation of COTS outbreaks. Hence, synchronized sequential outbreaks in separate islands are likely caused by similar environmental conditions, not by larvae traveling vast open ocean distances. Given the increased reef degradation projected for the near future due to anthropogenic activities [39, 40], COTS outbreaks could continue to increase in the following decades, as predicted by the degraded reef hypothesis [21]. However, since our results suggest that outbreaks are likely sourced from local COTS populations, preventing or minimizing COTS outbreaks might still be possible through local management plans. Our recommendation is to develop integrated, science-based management plans aiming to reduce reef degradation, improve early outbreak detection, and avoid the phytoplankton blooms that may trigger COTS outbreaks.

## Conclusions

Overall, our study suggests that crown-of-thorns seastar (COTS) outbreaks in the Pacific Ocean arise from locally derived populations rather than spreading across large open ocean distances. By analyzing 247 low-coverage whole-nuclear genome sequencing datasets, we resolved long-standing debates surrounding the dispersal dynamics of these devastating coral predators, demonstrating significant genetic structuring among geographically distinct lineages restricted to Hawai‘i, French Polynesia, and the West Pacific. Our findings further challenge the current taxonomic classification within *Acanthaster*, suggesting the presence of cryptic speciation within Pacific COTS populations: the Hawai‘i and the French Polynesia COTS lineages are particularly distinctive and likely represent yet-to-be-described species, while the species status of *Acanthaster* cf. *ellisii* is not supported by our phylogenetic results.Additionally, our demographic analyses reveal unprecedented recent expansions in COTS effective population sizes, and increased outbreak frequency and magnitude. The significant population structure and locally derived nature of outbreaks uncovered here stress the importance of local management strategies aimed at mitigating reef degradation and controlling anthropogenic stressors. Ultimately, this work underscores the substantial improvements whole genome approaches offer in unraveling complex ecological, evolutionary, and conservation-related questions, providing critical insights for reef management practices across the Pacific Ocean.

## Methods

### Study area and data processing

Raw reads were downloaded from the NCBI SRA BioProjects PRJNA548418, PRJDB10499, PRJDB9937, which included transcriptomic data for one *Acanthaster brevispinus* individual and genomic data for two *Acanthaster benziei*, two *Acanthaster planci*, 241 *Acanthaster* cf. *solaris*, and three *Acanthaster* cf. *ellisii* organisms. *Acanthaster* spp. individuals were collected from 26 sampling sites from the Red Sea, the North Indian Ocean, and the Pacific Ocean (Fig. 1A). The data were generated using different Illumina sequencing platforms and different read lengths (see details in [27, 41]). Hence, in order to avoid batch effects due to the use of different Illumina instruments, the data were processed following the guidelines from [42] and [43]. Raw reads were processed with fastp v.0.23.2 [44] using a sliding window approach with a phred score quality threshold of 33 (–cut-right -q 33), enabling adapter sequence detection for both reads (–detect_adapter_for_pe), deduplicating reads (–dedup) and finally trimming all reads to a length of 135 bp (–max_len1 135). Clean reads were then mapped against the *A.* cf. *solaris* reference genome (GCF_001949145.1_OKI-Apl_1.0; [28]) using bwa-mem2 v.2.2.1 [45]. BAM files were sorted and quality filtered (-q 20) with samtools v.1.10 [46] and overlap-clipped using bamUtil v.1.0.15 [47]. Mapping quality and depth were checked for each individual BAM file using qualimap v.2.2.1 [48]. After removing samples with low mean depth, the average depth across samples was 9.5 ×. Having this medium coverage dataset, we decided to prioritize the use of software that are based on genotype likelihoods, as recommended for low-to-medium coverage datasets [49] (see details below). However, we also used software based on genotype calls when appropriate, following other examples of population genomic studies using medium coverage datasets that combined both analytical approaches (e.g., [50–53]). Custom scripts are provided in a GitHub repository (https://github.com/cleivama/COTS-WGS-popgen) and archived in Zenodo (https://zenodo.org/records/15302534).

### Contamination detection

Preliminary analyses of population structure, relatedness, and heterozygosity suggested cross-contamination among some *A.* cf. *solaris* samples from the West Pacific. Hence, in order to detect and remove contaminated samples, contamination levels in all *A.* cf. *solaris* samples were estimated using verifyBamID v.2.0.1 with default parameters [54]. A total of 115,277 SNPs from the longest scaffold of the reference genome were used for these analyses, with a contamination threshold of 3% following authors’ recommendation [54]. Nine samples were removed from the dataset due to their high contamination levels (Additional file 2: Table S2).

### Population genetic structure, diversity, and inbreeding in the Pacific COTS *Acanthaster* cf. *solaris*

For the population genetic structure analyses, those individuals with mean depth lower than 4 × were removed to avoid depth biases. However, for three sites that had low sampling sizes (Hawai’i, Papua New Guinea, and the Philippines), those individuals with mean depth higher than 2 × were also included in these analyses, reaching a total of 198 individuals. The three samples from the Gulf of California were excluded from these analyses as they presented extremely low mean depth (0.3 × –1.3 ×).

Variant detection was performed using ANGSD, as it takes genotype uncertainty into account, as recommended for low to medium depth data. ANGSD was run with a bamlist including the 198 BAM files and the following options: -uniqueOnly 1, -remove_bads 1, -only_proper_pairs 1, -trim 0, -C 50, -minMapQ 20, -minQ 20, -doCounts 1, -GL 2, -doGlf 2, -doPost 1, -doMajorMinor 1, -doMaf 1, -doBcf 1, -doGeno 2, -SNP_pval 1e-6, -minMaf 0.05. The minimum number of individuals required to keep a site was calculated as 80% of the total number of individuals (198*0.8 = 158; -minInd 158). The minimum overall depth was set to twice the number of individuals (198*2 = 392; -setMinDepth 392) and the maximum overall depth was set to three times the overall mean depth across all individuals (9.5 ×) multiplied by the number of individuals (3*9.5*198 = 5643; -setMaxDepth 5643), following [55]. Then, the resulting genotype likelihoods file in bcf format was thinned using vcftools v.0.1.16 [56] to ensure a minimum distance of 10,000 bp between SNPs and was subsequently transformed to genotype likelihoods in beagle format using ANGSD with the following parameters: -doGlf 2, -doMajorMinor 1, -doMaf 1. The dataset for the West Pacific lineage, which included 140 samples from Vietnam to Fiji, was obtained by filtering the bcf file from the first ANGSD run using bcftools v.1.10.2 [46], thinning it with vcftools to ensure a minimum distance of 10,000 bp between SNPs, and transforming it to genotype likelihoods in beagle format using ANGSD with the same parameters detailed above for the whole dataset. VCF files from classic genotype calling, genotype likelihood calls from ANGSD, and mapping samples to populations are provided in Zenodo, complete with a detailed description for each file (https://zenodo.org/records/15302534).

Population genetic structure was assessed for both the whole dataset and the West Pacific dataset with PCAngsd v.1.10 [57] using the thinned datasets of genotype likelihoods in beagle format. Covariance matrices from PCAngsd were then used to compute eigenvalues and eigenvectors using the eigen function in *R* [58]. Results were plotted with ggplot2 [59]. PCAngsd was also used to compute admixture proportions for the whole dataset using the –admix option, which resulted in an optimal *K* value of 5. Admixture proportions for *K* = 3 and *K* = 4 were also calculated using the –admix_K option. Admixture proportions for the optimal *K* value were plotted as pies on a map using the mapmixture v.1.1.3 [60] R package. Additionally, a neighbor-joining tree was estimated for the whole dataset using the –tree option on PCAngsd, and plotted with FigTree v.1.4.4 (http://tree.bio.ed.ac.uk/software/figtree/). Pairwise *F*_ST_ indices were calculated between pairs of sampling sites to estimate their genetic differentiation, using the thinned vcf file containing genotype calls. The read.vcfR function in the vcfR package [61] was used to input the vcf file into *R*, where a pairwise *F*_ST_ table was calculated using the stamppFst function in the StAMPP R package [62] with 1000 bootstraps to calculate *p*-values. Sampling sites from Japan (Okinawa and Kagoshima) and French Polynesia (Bora Bora, Mo‘orea, Raiatea, and Tahiti) were analyzed independently for this analysis to test whether they present significant genetic differences.

Genetic diversity measures were calculated for each Pacific COTS lineage separately (i.e., West Pacific, French Polynesia, and Hawai’i). First, ANGSD was run independently for each lineage using the same options detailed above, adding the -doSaf 1 option to estimate the site allele frequency likelihood based on individual genotype likelihoods [63]. Then, a folded site frequency spectrum (SFS) was obtained for each lineage using the realSFS module within ANGSD, followed by the calculation of the thetas for each site as a measure of genetic diversity using the realSFS module with the saf2theta option. Finally, thetas per site were transformed to thetas in non-overlapping sliding windows of 50,000 base pairs using the thetaStat module within ANGSD with the following options: do_stat -win 50,000 -step 50,000. Raw thetas were divided by the total number of SNPs for each window to obtain corrected thetas following [64], which were then plotted by lineage with ggplot2. Differences among lineages were first tested with ANOVA using the aov function in R, followed by a Levene’s test to assess for homogeneity of variance across groups using the leveneTest function in the car R package [65]. Due to the significant differences of variance across groups, a Welch one-way test was performed using the oneway.test function in R, as it does not assume homogeneity of variance across groups. Similarly, differences between group pairs were tested using a pairwise Mann–Whitney *U* test using the pairwise.wilcox.test function in R, as it does not assume homogeneity of variance across groups. Multiple testing was controlled using a Benjamini-Hochberg (BH) correction and an adjusted *p*-value cut-off of 0.05.

Identity-by-descent regions throughout the genome (IBD tracks) were estimated per individual using ngsF-HMM v.1.1.0 [66], which takes into account the uncertainty of the data as it uses genotype likelihoods as input. Due to the existence of strong population structure in the whole dataset, ngsF-HMM was run for each of the three main lineages independently following ngsF-HMM’s authors’ suggestions [66]. Each independent run was launched using the ngsF-HMM.sh script included with the software, which runs 20 replicates of ngsF-HMM and chooses the best run, with the aim of avoiding convergence to local maxima, and results were plotted with ggplot2. Differences among lineages and between group pairs were tested as detailed above for the genetic diversity. Similarly, Welch one-way test and Mann–Whitney U test were used due to the significant differences of variance across groups.

Linkage disequilibrium (LD) decay was calculated per lineage using PopLDdecay v.3.42 [67] with default parameters to calculate the square of the correlation coefficient (*r*^2^) between pairs of loci, plotting the results using the Plot_MultiPop.pl perl script included in the package. Finally, relatedness indices were calculated for each pair of individuals in the thinned whole dataset using vcftools –relatedness2 option [68]. Both PopLDdecay and vcftools used genotype calls as input for these analyses.

### Demographic history of the Pacific COTS *Acanthaster* cf. *solaris*

Two different approaches were used to estimate historical changes of effective population sizes (*N*e) per lineage: an ancient estimation based on the SFS using StairwayPlot2 v.2.1 [69] and a recent demographic history inference based on LD using the Genetic Optimization for *N*e Estimation (GONE) [70]. Blueprint files for StairwayPlot2 were prepared with the following parameters: μ = 9.13e − 9 (nuclear mutation rate for the Pacific Ocean COTS; [71]); year_per_generation = 3 (generation time assumed for the Pacific Ocean COTS; [41]); and using the folded SFS of each lineage generated with ANGSD and realSFS (see the previous section).

For the GONE analyses, we first filtered the first and longest 199 scaffolds for each vcf file generated by ANGSD using bcftools, as it is the maximum number of scaffolds the software can analyze. The first 199 scaffolds contained 81.16% of the total genome length. Then, bcf files were converted to PLINK BED, FAM, and BIM format using PLINK v.1.9 [72], and were subsequently pseudo-haploidized using correctKin [73], as recommended for low-to-medium depth data [70]. Pseudo-haploidized files were converted to PLINK MAP and PED format using PLINK, and MAP files were modified using custom bash commands to generate input files for GONE (i.e., numerical scaffold names, separated by spaces instead of tabulations, and only allowing alphanumeric values in the SNP IDs). Analyses were run using the recommended values for our type of data [70]: using the default recombination rate of 1 cM/Mbp, indicating that input files were pseudo-haploidized (PHASE = 0), using all scaffolds (maxNCHROM =  − 99), analyzing 5000 random SNPs per scaffold in order to analyze approximately 1 million SNPs per run (maxNSNP = 5000), using the recommended maximum value of recombination rate analyzed of 0.05 (hc = 0.05), running 40 replicates per run (REPS = 40), removing SNPs with zeroes (ZERO = 0), and leaving the default values for the rest of the parameters. GONE was run 20 times for each lineage, with each run taking a new subsample of 5000 SNPs per scaffold, to calculate confidence intervals. StairwayPlot2 and GONE results were plotted together with ggplot2.

### Signals of selective sweeps

Genomic signals of hard selective sweeps were explored for each lineage using the Raised Accuracy in Sweep Detection (RAiSD) v.2.9 method [74]. In order to simplify the final Manhattan plots, RAiSD was run using the filtered vcf files that included the first 199 scaffolds. Input vcf files contained genotype likelihoods, which are internally transformed to genotype calls by RAiSD. RAiSD was run with default parameters and the options -P to generate four plots for each scaffold and -A 0.9995 to generate the final Manhattan plots using a probability value of 0.9995 for the quantile function in R. The highest peaks were annotated using the genome annotations from the *A.* cf. *solaris* reference genome [28].

### Phylogenetic analyses

A phylogenomic approach was used to investigate the relationships among the different COTS species and lineages. First, ANGSD v.0.938 [75] was run with 204 individuals including two *A. benziei* from the Red Sea, two *A. planci* from the North Indian Ocean, 198 *A.* cf. *solaris* from the Pacific Ocean, and two *A.* cf. *ellisii* from the Gulf of California. ANGSD was run with -doHaploCall 2 -doCounts 1 -minMinor 1 to call pseudohaploid SNPs on the basis of sampling the most common base for each site, in order to minimize bias due to low depth following [76]. The resulting.haplo file was filtered to only include those SNPs present in at least 90% of the samples and transposed into a fasta alignment using custom R scripts. Both the R scripts and the fasta file are publicly available in Zenodo (https://zenodo.org/records/15302534). Maximum likelihood analyses were conducted using IQ-TREE v.2.3.4 [77] with 1000 bootstrap replicates and the GTR + ASC model as recommended for SNP data. In order to properly root the tree, a second ANGSD run was performed with the same parameters as described above to call pseudohaploid SNPs (-doHaploCall 2 -doCounts 1 -minMinor 1), using transcriptomic data from the closest COTS outgroup, *Acanthaster brevispinus*, and one individual representative of each COTS species and lineage. The resulting file was filtered to only include SNPs present in all samples and transposed into a fasta alignment, which was used as input for IQ-TREE with 1000 bootstrap replicates and the GTR + ASC model.

## Supplementary Information

Additional file1: Table S1.

Additional file2: Table S2.

Additional file3: Fig. S1.

## Data Availability

No new sequencing data were generated for this study. Raw sequencing data analyzed during the current study are available in the NCBI repository, BioProjects: PRJDB10499 (https://www.ncbi.nlm.nih.gov/bioproject/?term=PRJDB10499), PRJDB9937 (https://www.ncbi.nlm.nih.gov/bioproject/?term=PRJDB9937), and PRJNA548418 (https://www.ncbi.nlm.nih.gov/bioproject/?term=PRJNA548418). Additional file 1: Table S1 includes the NCBI BioProject, BioSample and SRA Run for each individual COTS sample analyzed. VCF files from classic genotype calling, genotype likelihood calls from ANGSD, mapping samples to populations are provide at Zenodo, complete with a detailed description for each file (https://zenodo.org/records/15302534). Custom scripts are provided in GitHub (https://github.com/cleivama/COTS-WGS-popgen), and a release at the time of publication has been archived in Zenodo (https://zenodo.org/records/15302534). NCBI Bioproject Accession PRJDB10499. Pandemic Outbreak of Crown-of-Thorns Starfish (COTS) Populations in the Pacific Ocean Description. Marine Genomics Unit, Okinawa Institute of Science and Technology. 2022. https://www.ncbi.nlm.nih.gov/bioproject/?term=PRJDB10499. NCBI Bioproject Accession PRJNA548418. Acanthaster brevispinus. University of the Sunshine Coast. 2019. https://www.ncbi.nlm.nih.gov/bioproject/?term=PRJNA548418. NCBI Bioproject Accession PRJDB9937. Comparative genomic analysis among Acanthaster spp. School of Life Science and Technology, Department of Life Science and Technology, Tokyo Institute of Techonology. 2021. https://www.ncbi.nlm.nih.gov/bioproject/?term=PRJDB9937. Zenodo repository with datasets and scripts. Acanthaster whole genome datasets and scripts. Carlos Leiva. 2025. 10.5281/zenodo.15302534.
