# Supplementary material for: Genomic analyses support locally derived crown-of-thorns seastar outbreaks in the Pacific
Source: BMC Biol. 2025 Aug 6;23:244. doi: 10.1186/s12915-025-02350-4 (PMC12330192; doi:10.1186/s12915-025-02350-4)
Supplement: Supplementary file 3 — Additional file3: Fig. S1. [file 12915_2025_2350_MOESM3_ESM.pdf]

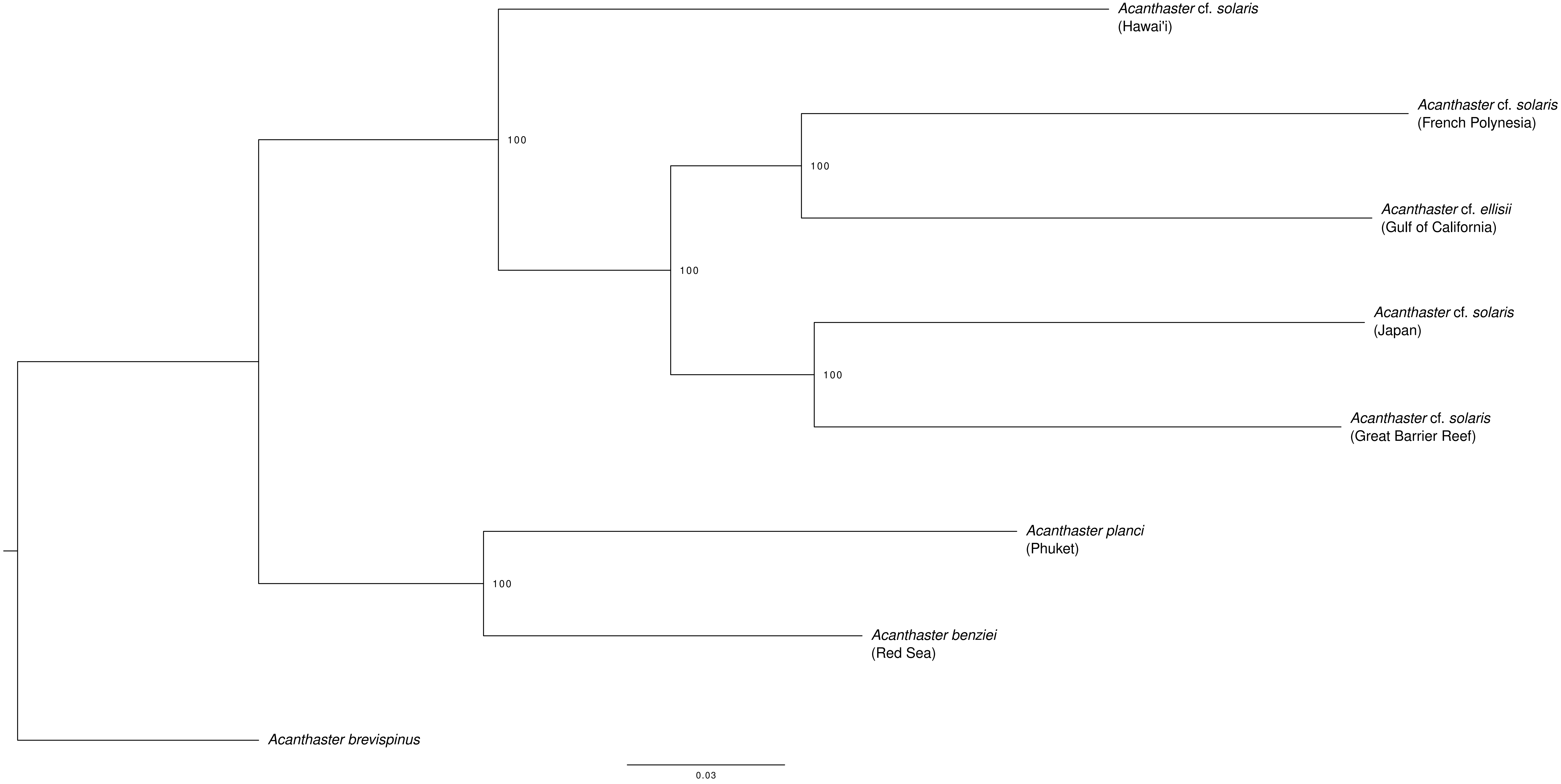

Additional file 3: Fig. S1: Maximum likelihood tree including one representative of each COTS species and lineage, with 46,438 genome-wide SNPs shared by all samples. This result robustly supports that *A. benziei* and *A. planci* are sister species.
